# Bidirectionality and Compartmentation of Metabolic Fluxes Are Revealed in the Dynamics of Isotopomer Networks

**DOI:** 10.3390/ijms10041697

**Published:** 2009-04-17

**Authors:** David W. Schryer, Pearu Peterson, Toomas Paalme, Marko Vendelin

**Affiliations:** 1 Laboratory of Systems Biology, Institute of Cybernetics, Tallinn University of Technology, Akadeemia 21, 12618 Tallinn, Estonia; E-Mails: david@sysbio.ioc.ee (D.W.S.); pearu@sysbio.ioc.ee (P.P.); markov@sysbio.ioc.ee (M.V.); 2 Department of Food Processing, Tallinn University of Technology, Ehitajate 5, 19086 Tallinn, Estonia; E-Mail: tpaalme@staff.ttu.ee (T.P.)

**Keywords:** Metabolic network, isotopomer dynamics, MFA, mathematical modeling, compartmentalization, ^13^C NMR

## Abstract

Isotope labeling is one of the few methods of revealing the *in vivo* bidirectionality and compartmentalization of metabolic fluxes within metabolic networks. We argue that a shift from steady state to dynamic isotopomer analysis is required to deal with these cellular complexities and provide a review of dynamic studies of compartmentalized energy fluxes in eukaryotic cells including cardiac muscle, plants, and astrocytes. Knowledge of complex metabolic behaviour on a molecular level is prerequisite for the intelligent design of genetically modified organisms able to realize their potential of revolutionizing food, energy, and pharmaceutical production. We describe techniques to explore the bidirectionality and compartmentalization of metabolic fluxes using information contained in the isotopic transient, and discuss the integration of kinetic models with MFA. The flux parameters of an example metabolic network were optimized to examine the compartmentalization of metabolites and and the bidirectionality of fluxes in the TCA cycle of *Saccharomyces uvarum* for steady-state respiratory growth.

## 1. Introduction

Isotope labeling is widely used to gain insight into the operation of metabolic networks despite the fact that neither the collection of isotopomer data, nor its simulation and analysis is considered routine. Both experimental and analytical methods enabling dynamic studies that require direct measurement of the mass and/or positional isotopomers and the pool sizes of intermediate metabolites are developing quickly [1, 2, 3]. The move from isotopomeric steady state flux analysis to studies involving dynamic enrichment is required to deal with the complexities of the eukaryotic cell and multicellularity. The compartmentalization of metabolites into organelles, often with parallel enzyme systems coupled with complex transport mechanisms makes the application of Metabolic Flux Analysis (MFA) at isotopic steady state difficult and uncertain.

MFA is an important tool for strain improvement in biotechnology [4] with a vast potential for further improvement. However it has recently been stated that ”in order to truly exploit the synthetic capacity of biological systems and broaden the creation of microbial chemical factories, it is necessary to go beyond natural pathways for the synthesis of natural products towards the *de novo* design and assembly of biosynthetic pathways for both natural and unnatural compounds.” [5]. Synthetic Biology, while probable in the long term, is optimistic in light of our current understanding of metabolic systems and will depend on knowledge gained from the flux analysis of natural pathways. The great potential for genetic improvement has not been realized largely due to an incomplete understanding of the metabolic operation within organisms - especially their dynamic nature.

This paper is a short review of the motivations for moving from MFA using data collected at isotopic steady state to making full use of the information contained in the isotopic transient. Examples are taken from recent studies that make good use of this information followed by a short section on performing this analysis under conditions of unstationary metabolism. An attempt is made to point towards the future of dynamic modeling of cellular systems using predictive kinetic models–The holy grail of modern biology. Simulations of isotopic transients are used to explore the information contained in the isotopic transient and examine techniques to exploit this information. Following this is a short example where the flux parameters are optimized for the TCA cycle in *Saccharomyces uvarum* for steady-state respiratory growth fed with ^13^C_1,2_ acetate and unlabeled glucose.

### 1.1. Motivation for exploiting the dynamic transient

The majority of MFA studies have been conducted at metabolic steady state, and the majority of these involve measuring isotopomers at isotopic steady state. Recent studies conducted at the metabolic and isotopic steady state include Blank *et al.* [6] and Vo *et al.* [7]. These and other studies have contributed and will continue to contribute to our understanding of metabolic function, however MFA at metabolic and isotopic steady state is complicated by a number of factors including compartmentalization [8, 9] and makes it more difficult to study the robustness of metabolic networks [10] since a separate flux analysis is required for each metabolic perturbation. Dynamic isotopic analysis allows one to directly probe metabolic robustness and control.

A recent study demonstrates the use of MFA at metabolic steady state using isotopic transient data in the pentose phosphate pathway and citric acid cycle (TCA) of *E.coli* [11]. Their modeling was made easier by assuming that the flux from precursor metabolites to free amino acids to protein bound amino acids was uni-directional and there was no lag in the isotopomer dynamics due to protein turnover and bi-directional transamination reactions as measured in *Saccharomyces cerevisiae* [12]. den Hollander *et al.* [13] measured this effect in 1981 using ^13^C NMR to track metabolite dynamics. Although little is known about protein turnover rates *in vivo* prokaryotes are expected to display less protein turnover than eukaryotes [14]. Isotopic dynamics in prokaryotes avoids the most obvious types of compartmentalization, so most examples of MFA in this review are taken from eukaryotic systems.

## 2. Dynamic MFA in eukaryotic systems

MFA using isotopic transient data is more often applied in eukaryotic systems as it is not so easy to avoid compartmentalization and bi-directional exchange with large metabolic pools. However, since the nature of many of these dynamic processes has yet to be elucidated, MFA using isotopic transient data has been performed mostly on small linear branches of the metabolic networks without accounting for global dynamic behavior [15]. There are a few exceptions however, notably Heinzle *et al.* [16] who used a combination of kinetic network modeling and simulation to calculate metabolic fluxes in a secondary metabolic network in potato (*Solanum tuberosum*). Shastri and Morgan [17] assess the experimental needs for conducting isotopic transient MFA experiments on plants, and a few recent papers review techniques for determining fluxes in plant networks [18, 19].

Often, the organism of interest cannot be sustained in a steady metabolic state over long periods of time. To overcome this limitation one could resort to simulating the isotopic transient with a non-steady metabolism, or shorten the labeling experiment to less than one minute since the concentrations of enzymes in cells remain constant over short time spans (10 s to 1min) [20].

### 2.1. Flux analysis with non-steady metabolism

There has been some progress recently in MFA studies with a non-steady metabolism and a lack of kinetic structure. A few researchers have started the move towards non-stationary MFA, with Wahl *et al.* [21] and Baxter *et al.* [22] recently publishing papers that outline frameworks for performing transient isotopic experiments under a transient metabolic state. Experimental and analytical techniques have advanced to the point where it is possible to collect the data needed for studies involving non-steady metabolism, and this class of dynamic MFA should start becoming more common and will aid in excluding hypotheses regarding cellular compartmentalization and dynamic metabolic behavior.

### 2.2. Utilizing metabolic oscillations

It is widely accepted that metabolic systems ubiquitously display oscillations in metabolic fluxes through temporal compartmentalization, proposed to be driven by oscillating metabolic cycles [23]. By turning metabolic cycles on and off biochemical reactions can be carried out under optimal conditions and futile cycles reduced. Fluctuations in fluxes have prompted Wiechert and Noh [14] to argue that “MFA is currently reaching the biological limits of its applicability” because population inhomogeneities and flux oscillations prevent one from obtaining meaningful dynamic measurements. There are cases when these limitations can be minimized through the use of oscillations, however.

In continuous culture yeast can be enticed to grow with a stable oscillating metabolism with a period between 40 minutes to 5 hours[24]. While growing in this state the metabolic state of most cells in the fermenter are operating in synchrony, thus reducing population inhomogeneities to a minimum, although it should be noted that some inhomogeneities persist, such as that due to cells operating at different stages in the cell cycle. Tu *et al.* [25] measured the periodicities of expressed genes while yeast was growing in this state and found that over half of the (≈ 3,552) yeast genes exhibited periodic expression at a confidence level of 95%. Tu *et al.* [25] conclude by arguing that metabolic oscillation may ”constitute the primordial device upon which the divergent circadian and ultradian biological oscillators of modern organisms have been built”.

Keeping in mind that enzyme concentrations remain constant over short time spans it is conceivable that one could use a device like the BioScope [26] to perform transient isotopic pulse experiments at different stages in the oscillating cycle (at a good approximation to metabolic steady state over the sampling period) thus avoiding metabolic inhomogeneities in the vast majority of the population and large flux oscillations. This would enable the analysis of metabolic fluxes using isotopic transient data at different metabolic states under one cultivation condition. A data set of this nature could also be used for MFA at the metabolic and isotopic steady state and could aid in the construction of a predictive large scale kinetic model of yeast metabolism with cell signaling dynamics [27].

## 3. Building predictive kinetic models

Predictive kinetic models can be created in systems where the *in vivo* kinetics of many enzyme systems within the metabolic network are well characterized. For many systems this information is not available, so development of kinetic models of metabolic systems is much less common than the use of phenomenological MFA to characterize metabolic activity. However, predictive kinetic models allow us to use the information content of experimental data points measured at one physiological condition to predict the dynamic behavior of the system at another physiological condition.

The modeling process involves (1) developing a theory of how the biological system operates, (2) representing the system as a set of ordinary and/or partial differential equations with direct physical meaning, (3) fitting the parameters of this system using one dataset, (4) testing the predictive qualities of the system using another related dataset, and (5) adjusting the theory and repeating the process as required. Metabolic models that have passed this kind of scrutiny allow us to predict bi-directional metabolic fluxes and system behavior under conditions where measured data is sparse. Great improvements can be achieved with the use of data gathered decades ago, which is often of high quality and fundamental in nature.

The complexity and scope of the model ought to be limited by the quality and amount of measured data used to tune it, so introduction of kinetic parameters into dynamic models must be carefully considered. It is wise to restrict the addition of kinetic parameters to enzyme systems that have been systematically studied such that the kinetic scheme is biologically relevant and the kinetic parameters are known with some level of confidence. This ensures that there is additional data available for the tuning process, and the parameters are physiologically relevant.

With this approach it is possible to maintain the structural identifiability of the model while adding more parameters. If many parameter sets can fit the available data, biological insight is severely limited if not impossible, so it is wise to always check the robustness of the solution during parameter optimization. With this in mind, it is not recommended to replace phenomenological MFA with phenomenological kinetic schemes that include more parameters since this only works to reduce the structural identifiability of the model while adding no biological insight.

Ultimately, the construction of a predictive kinetic model involves the laborious task of studying each enzyme system *in vivo* under a wide range of metabolic conditions. With the availability of additional kinetic insight and data metabolic flux analysis in the heart has progressed along a different path from the microbial and plant systems mentioned above. Predictive kinetic models in the heart are widespread since drug development is only possible with fundamental knowledge of enzyme operation, and this work is best performed in the public domain. With the future shift towards the use of cellulosic biorefineries it is predicted that there will be an increasing economic stimulus to study the fundamentals of exotic metabolisms and thus a resurgence in fundamental kinetic studies in plant and microbial systems.

With the complexity of biological systems, predictive models are useful to exclude hypotheses regarding their function. Vendelin *et al.* [28] quantified the oxygen dependence on the workload in rat cardiomyocytes using published data. By working with the kinetic assumptions in the model they refuted the assumption that the ADP concentration does not contain gradients, and found the gradients to be workload-dependent. Intra-cellular concentration gradients were not required for phosphocreatine, creatine, and ATP, whose concentrations can be assumed to be in spatial equilibrium. The change in ADP concentration taken together with changes in inorganic phosphate were found to be major components of the metabolic feedback signal to control respiration in muscle cells. Using the same modeling approach, the control of respiration was found to be dependent on the dynamics of the system [29].

Predictive kinetic models are better suited to exclude hypotheses regarding dynamic metabolism than phenomenological MFA. Selivanov *et al.* [30] and Liebermeister and Klipp [31] have published methods to make use of transient isotopic data in predictive kinetic models of dynamic cellular behavior, although the application of this technique is in its infancy due to the complexities of the underlying dynamic system including the problem of how to analyze multi-compartment labeling. The use of kinetic information coupled with isotopomer analysis will become an increasingly important tool.

### 3.1. Measurement of in vivo kinetics

One important tool for probing the mechanisms of complicated kinetic systems *in vivo* is the NMR saturation and inversion transfer technique developed in theory by McConnell [32] and in practice by Forsen and Hoffman [33]. Nuclei having been saturated or inverted with radio frequency radiation can retain their magnetic orientation through a chemical reaction. Thus, if the time span of the reaction is short compared to the relaxation time, the NMR spectrum may show the effects of the saturation or inversion on the corresponding, unirradiated line in the spectrum. Saturation and inversion detects only the pool of molecular species that are able to react, and gives direct insight into reaction kinetics and metabolite compartmentalization.

A number of reviews discuss techniques for using saturation and inversion transfer for studying the kinetics of complex reaction schemes [34, 35, 36]. The application of saturation and inversion transfer using ^31^P NMR to study the energy metabolism in hearts is a good example of how compartmentalization and bi-directionality of reaction steps complicates the analysis of a small network of reactions.

Early studies observed a discrepancy between the measured forward and reverse rate in the creatine kinase reaction when the myocardium was operating at steady state. To resolve this discrepancy it was concluded that analysis of the NMR data should include either compartmentalization of substrates or enzymes, or include an exchange of ATP with other phosphorus species such as inorganic phosphate [37, 38]. In the case of compartmentalization, each compartmentalized pool will require fitting a different *T*_1_ value [39].

Since the amount of information available from one single magnitization transfer protocol is insufficient to fit all parameters, Joubert *et al.* [40] used four different magnitization transfer protocols in one experiment and used this additional data to fit multiple possible kinetic schemes. They determined that three different creatine kinase reactions schemes should be considered and both subcellular compartmentalization and multiple exchange with inorganic phosphate are important. This work reveals insight into the spatial and temporal buffering of ATP in cardiac cells [41], which is linked with heart failure when operating in a sub-optimal mode [42].

A complimentary method for exploring *in vivo* kinetics was developed to study energy metabolism in skeletal muscle using mass spectrometry to follow the enrichment of oxygen isotopes into energy metabolites. Replacing the external cellular environment with ${\text{H}}_{2}^{18}\text{O}$ results in the incorporation of hydroxyl ions from ${\text{H}}_{2}^{18}\text{O}$ into the phosphoryl groups of energy metabolites resulting in an equilibrium distribution of phosphoryls with 1, 2, or 3 ^18^O atoms as a function of the enrichment of ^18^O in the water [43]. The size of metabolic pools can be calculated from the distribution of these molecular species at isotopic equilibrium, and using the time course of ^18^O incorporation into the high-energy phosphoryls one can determine the rate of hydrolysis of the energy metabolites [43].

There are a number of technical difficulties when implementing this approach. The analytical work is very laborious and many animals are required for a statistically significant study. Each dynamic data point requires sacrificing one animal where an ^18^O transient is induced, followed by freeze clamping in liquid nitrogen and a long preparatory procedure prior to analysis in the mass spectrometer.

The analysis of the data is also tricky since phosphotransfer dynamics contain compartmentalized metabolites and bi-directional reaction steps. To simplify the analysis of their transient experimental data on the uptake of ^18^O in the energy metabolites of toad skeletal muscle, Dawis *et al.* [43] assumed that the fluxes through the enzymatic complexes were uni-directional and only one ^18^O could be incorporated per molecular turnover. They judiciously discussed the issues bi-directional reaction steps within enzymatic complexes and wrote that “In practice, it will be difficult to verify a multiple-reversal model for the intact cell. Consequently, it will not be easy to choose between a multiple reversal model and a compartmentalization model.” Dawis *et al.* [43] also stressed that the influence of bi-directional reaction steps “should be examined but will be difficult to prove.”

A proper study of the bi-directionality of phosphotransfer networks has yet to be completed, and the amount of data collected in ^18^O transfer studies is probably not enough to distinguish between possible reaction networks with various combinations of compartmentalization and bi-directional fluxes. Because of these limitations the above assumption of uni-directional fluxes was applied in a series of papers that explored the kinetics and compartmentalization of energy metabolism in rat skeletal muscle [44, 45, 46, 47, 48]. However, the assumption of uni-directional fluxes is not a necessary limitation of the method and should be evaluated in future studies.

Saturation and inversion using ^31^P NMR can be enhanced by the use of either a ^17^O or ^18^O induced isotope shift in the ^31^P NMR spectra. Pucar *et al.* [49] introduced the ^18^O assisted ^31^P NMR method to study energetics in mouse heart. The method was employed in a series of papers exploring compartmentalized energetics [50, 51, 52] [53, Pages 178–181], with each study using the above mass spectroscopy method to determine longer time ^18^O transfer kinetics, all with the same assumption of uni-directional fluxes. The development of improved methods utilizing NMR saturation and inversion will extend the range of applicability of this powerful technique [54, 55] while reducing the labor required.

## 4. Simulation of isotopic transients

The isotopic transient contains information about the underlying behavior of the metabolic system. The task is to build a model of the metabolic system that can best reproduce both the isotopic transient and the steady state isotopomer distribution of all metabolites. This involves finding the sizes of metabolic pools, the bi-directional rates of exchange between compartments in the cell, and the effect of bi-directional enzyme reactions on the isotopomer distribution. Of these, only the sizes of metabolic pools do not affect the steady state labeling state of the metabolites and the biomass created from them.

### 4.1. Composition of the metabolic network

**Figure 1.**
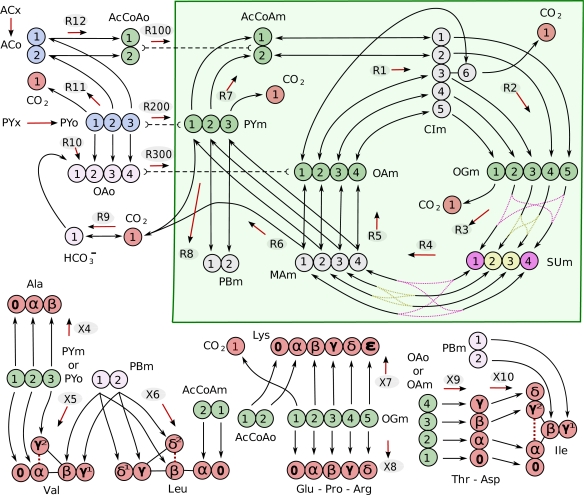
Metabolic scheme with atom mapping and bi-directional compartmentalization between mitochondria (shaded green) and cytosol. Carbon numbers correspond to chemical nomenclature and the arrows between them indicate bi-directionality. Each reaction label is given above the red arrows that indicate the assumed net positive reaction flux. Pyruvate (PYx) derived from extracellular glucose and acetate (ACx) are inflows to the system (blue), and CO_2_ and amino acids are outflows(red). Metabolite abbreviations are given in Table 1. Green carbons indicate biomass precursor metabolites with mappings to the amino acids they produce. Carbons of the same color are equivalent due to molecular symmetry.

To aid in the discussion of extracting information from isotopic transient data, we have composed a simple example of the TCA cycle with carbon enrichment found in Figure 1. Included in the metabolic scheme are atom mappings between all species including the amino acids and their respective biomass precursors, with the carbon numbers corresponding to chemical nomenclature as in Maaheimo *et al.* [56]. Pyruvate and acetate are inflows to the system, and carbon dioxide and biomass precursors are outflows. The metabolic system is assumed to operate at steady state and is thus simulated with net flux distributions that satisfy this criteria. There are eight degrees of freedom in this system, so eight net fluxes are specified. The remaining dependent net fluxes were calculated from equations that were generated symbolically.

Analogous schemes can be drawn for any biological isotope including oxygen, phosphorus, and nitrogen isotopes, although the atom transitions in these networks are less well defined and functional groups containing these elements tend to be more reactive resulting in a network with a significant number of side reactions and sinks that complicate analysis as in the above phosphotransfer network studies.

### 4.2. Solving for the isotopic transient state

Isotopomer balance equations can be generated from the metabolic network, and using these, an isotopic transient can be simulated. The transient is induced by a step change in any or all members of the isotopomer population distribution of all metabolites that act as inputs or outputs to the system. For isotopomers that act as outputs to the system, the bi-directionality of the exit reaction step will induce isotope labeling in reverse direction to the net flux. The isotopomer distributions of all metabolites in the system begin at the natural labeling state of 1.1% ^13^C and end at isotopic equilibrium at an enriched ^13^C state with steady isotopomer population distribution. Thus, the steady state isotopomer distribution for each metabolite is found from the last points of the simulation when the system has reached isotopic steady state.

We used the most direct approach to solve for the isotopic transient by numerically solving the full set of isotopomer balances. Various strategies have been devised to transform this system into an equivalent system that is computationally more efficient to solve, including the bondomer approach [57], decomposition of the network into Elementary Metabolite Units (EMU) [58], and transforming the isotopomer equations into cascaded cumomer systems [59] where lumped variables are used to represent groups of isotopomers. The 252 isotopomer balance equations in our small example network are solved in 0.4 to 6 seconds when setting the metabolic pool sizes as being equal, so use of the above methods to speed up simulation is not required in this case.

To illustrate the information one can obtain from the isotopic transient, we present two sets of simulations. Our nomenclature for isotopomers in the figures and discussion below can be summarized as follows: The carbons are numbered according to chemical nomenclature and start at the right with 0’s representing ^12^C and 1’s representing ^13^C.

**Figure 2.**
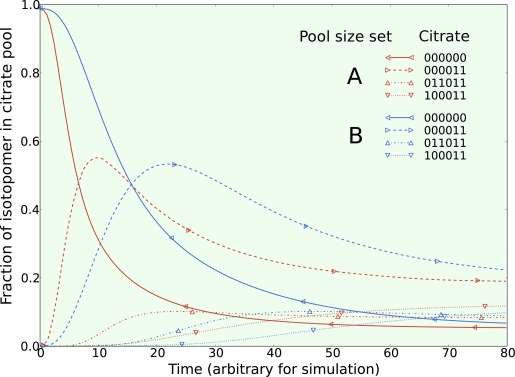
The isotopic transient of the metabolic system given in Figure 1 was simulated with two different sets of metabolic pool sizes chosen at random over three orders of magnitude. All other parameters are the same between the two simulations. For clarity, only the isotopomers of mitochondrial citrate reaching the highest enrichment are included with their nomenclature explained in the text.

*The first set* was obtained by continuously feeding pyruvate and acetate while performing a step change in the acetate isotopomer population from natural enrichment to 100% fully labeled ^13^C_1,2_ acetate. Two simulations were made with two different sets of metabolic pool sizes (A and B). The pool sizes of all metabolites in both sets were selected at random over three orders of magnitude. All net flux and exchange flux parameters were the same in both simulations. Since only metabolic pool sizes were changed between simulations, the steady state isotopomer distribution are identical for both simulations, as expected. The isotopic transients of the most highly enriched isotopomers of mitochondrial citrate from both simulations are given in Figure 2. Comparing the transient curves for the same isotopomers between pool size set A and B, it is clear that they exhibit the same general transient shape with the main difference being the time scale of the transient. Figure 2 does not show every isotopomer, however all carbons become enriched in ^13^C when acetate is used as the tracer illustrating the usefulness of this inexpensive tracer for studying the TCA cycle.

**Figure 3.**
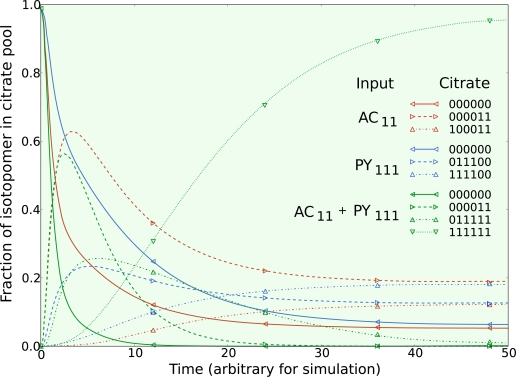
Three simulations of isotopic dynamics in the metabolic system given in Figure 1 were performed with identical net flux, exchange flux, and metabolic pool sizes. Isotopic transients of mitochondrial citrate are given following a switch to: (1) fully labeled acetate, (2) fully labeled pyruvate, and (3) both fully labeled acetate and pyruvate. For clarity, only the isotopomers of mitochondrial citrate reaching the highest enrichment are included.

*The second set* of simulations was obtained by continuously feeding pyruvate and acetate. The three simulations were made by performing (1) a step change to fully labeled acetate as above, (2) a step change from natural enrichment to 100% fully labeled ^13^C_1,2,3_ pyruvate, and (3) a step change in both fully labeled acetate and fully labeled pyruvate together. All other parameters, including metabolic pool sizes, net fluxes, and exchange fluxes were the same in all three simulations. The citrate isotopomers from these three simulations are given in Figure 3.

Different isotopomers from each of the three simulations display similar dynamics as the metabolic system is operating in the exact same way. Comparing the dynamics of the citrate isotopomers between the acetate and pyruvate switch, three pairs of isotopomers reach the same proportion of the steady state isotopomer population: (1) the unlabeled citrate isotopomers, (2) the 000011 and 111100 complimentary pair, and (3) the 100011 and 011100 complimentary pair. Different isotopic tracers reveal the same underlying metabolic behavior at steady state for the TCA cycle intermediates, with the dynamics revealing complimentary information.

When fully labeled acetate is fed to the metabolic system, the 000011 citrate isotopomer reveals similar dynamics as the same isotopomer when both acetate and pyruvate are fed to the metabolic system. When fully labeled pyruvate is fed to the metabolic system, the 011100 citrate isotopomer reveals similar dynamics as the 011111 citrate isotopomer when both acetate and pyruvate are fed to the system.

When both labeled acetate and labeled pyruvate enter the metabolic system, we see both types of isotopomer dynamics appear, however in this case when the isotopomer populations of pyruvate and acetate consist of 100% fully labeled compounds all information about the steady state is lost as the system becomes fully labeled. Thus the use of multiple labeling experiments on the same metabolic system under the same growth conditions is useful to study the dynamic behavior of the metabolic system, and is thus useful to gain insight into the metabolic pool sizes, compartmentalization, and the bi-directionality of metabolic fluxes.

To make these two example simulations quantitative one must find the appropriate metabolite pool sizes, net fluxes, and exchange fluxes that adequately reproduce a sufficient amount of transient isotopomeric data, possibly supplemented with additional steady state isotopomeric data, measurements of metabolic pool sizes, substrate utilization rates, and biomass production rates.

## 5. Extracting information from isotopomeric data

Any difference between measured data and model predictions can be used in an optimization routine to find sets of net fluxes, exchange fluxes, and pool sizes that can reproduce the measured data within experimental errors. If the optimization routine cannot obtain a realistic fit with a sufficient amount of data, the metabolic scheme must be adjusted, possibly with the inclusion of compartmentalization and the process repeated. After finding a set of model parameters that can sufficiently reproduce measured data, one can gain insight into the operation of the metabolic network.

All types of isotopomeric measurement can be compared with the output from the dynamic solver, including data collected at isotopic steady state: Mass isotopomers from mass spectrometers, NMR positional enrichments, double enrichments, triple enrichments, and beyond all contain information about the operation of the metabolic scheme. Each measurement type requires one to sum up the appropriate pool of simulated isotopomers that correspond to the measured ^13^C enrichment probability.

It should be noted that the process of optimization is not restricted to experiments performed with one enriched substrate. Data from multiple experiments at the same metabolic state using different labeled substrates can be combined to optimize one set of parameters. In this case the optimizer must simulate the isotopomer balance equations once for every experiment with a different step change in labeled substrate using the same set of parameters, and comparing each with their respective set of experimental data. The three simulations in Figure 3 could each be matched with data collected using labeled acetate, labeled glucose or a mixture of both to optimize the single set of parameters that govern the metabolic system.

### 5.1. Inclusion of metabolic pool sizes

Since it is difficult to accurately measure many metabolic pools, making the transient simulation quantitative typically requires additional transient isotopic data. Using an optimization routine it is possible to find a realistic set of metabolic pool sizes that best match isotopic transient data and pool size measurements. To accomplish this, the optimizer would be allowed to manipulate all metabolic pool sizes, thus changing the isotopic transient, while attempting to minimize the difference between measured isotopomeric data and measured pool sizes. In practice one would not usually optimize only the metabolic pool sizes as one usually needs to optimize the net flux and exchange flux parameters at the same time.

Figure 2 shows a dramatic increase and then decrease in the ^13^C_1,2_ isotopomer of citrate. With this in mind, transient data that is able to capture the shape and timing of major transient curves like this one are useful for constraining not only the net fluxes and bi-directionality of the metabolic network, but also metabolic pool sizes. If the pool size found by optimization does not match that measured during the experiment, it could be a clue that this metabolic pool is compartmentalized. Other clues in the shape of these transients also aid in identifying compartmentalization.

### 5.2. Compartmentalization is revealed in the dynamics

Information about the bi-directionality of fluxes and the compartmentalization of metabolic pools is contained in the isotopic dynamics. Compartmentalization is revealed in a number of ways. Consider a linear pathway:

(1) A⇌B⇌C

If the labeling in C becomes enriched faster than B, B is compartmentalized. This means that one should optimize the flux parameters for at least two separate pools of B:
(2)
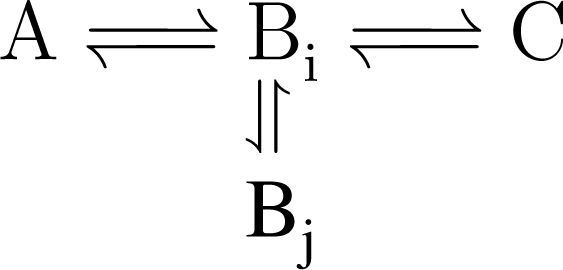


The shape of the isotopic transient depends on the exchange of B_i_ with B_j_ and their pool sizes. ATP exhibits compartmentalization in cardiomyocytes and astrocytes, as evidenced by a ^31^P NMR saturation and inversion analysis of the creatine kinase reaction[60]:

(3) $${\text{PCr}}^{2-}+{\text{MgADP}}^{-}+{\text{H}}^{+}⇌\text{Cr}+{\text{MgATP}}^{2-}$$

The kinetic data suggest that ATP exchanges with inorganic phosphorus and participates in other reactions via separate compartments:

(4) $$\text{PCr}\overset{k_{1f}}{\underset{k_{1r}}{⇌}}\gamma ATP1\overset{k_{2f}}{\underset{k_{2r}}{⇌}}\text{Pi}\overset{k_{3f}}{\underset{k_{3r}}{⇌}}\gamma ATP2$$

Fitting the data to this kinetic scheme suggests the need to consider both the function of the bound enzymes and restrictions of diffusion in the system, which both may lead to localized compartmentalization. Evidence for diffusional restrictions and compartmentalization of ATP was explored by Sonnewald *et al.* [61] who observed large gradients in ATP concentration in astrocytes. Monge *et al.* [62] performed a kinetic analysis of oxidative phosphorylation in rat brain synaptosomes and mitochondria and found evidence for localized cycling of ADP and ATP between mitochondrial creatine kinase and adenine nucleotide translocase.

Localized compartmentalization of energy metabolites in cells with high energy requirements is well known [63, 64]. Kaasik *et al.* [65] studied the energy metabolism in mouse cardiomyocytes and demonstrated that this localized cycling of energy metabolites was effective enough to maintain a moderate workload even in genetically modified mice deficient in creatine kinase. These studies clearly show the functional importance of localized compartmentalization separated by diffusional barriers. Furthermore, diffusional restrictions of ADP in rat cardiomyocytes could influence the control mechanisms of oxidative phosphorylation, as shown in several modeling studies [66, 67].

Vendelin and Birkedal [68] found diffusion coefficients in rat cardiomyocytes using a fluorescently labeled ATP analogue and found them to be anisotropic. For this, raster image correlation spectroscopy (RICS) was extended to discriminate anisotropy in the diffusion tensor. Although the reason for the anisotropic diffusion is unclear, it may be related to the ordered structure of the cardiomyocytes or localized diffusional barriers. To explore these localized diffusional barriers on a cellular level using mathematical models, the accurate geometry of mitochondria within the muscle cells is required. Vendelin *et al.* [69] developed a method to analyze the two dimensional positioning of mitochondria in various muscle types, and extended this method to three dimensions in a comparative physiology study between trout and rat cardiomyocytes [70].

Compartmentalized metabolic pools may play a role in controlling shifts in metabolism. Separate cytosolic pools of pyruvate in astrocytes have been observed to switch between acting as the precursor for energy production depending on the substrate being consumed [71]. In general compartmentalization is more complex than we have previously assumed and we may only be scratching the surface with regards to studying compartmentalized metabolism in cellular systems. With this view it is hard to avoid introducing realistic kinetic schemes into dynamic flux analysis.

### 5.3. Example optimization of the TCA cycle in yeast

To illustrate the process of extracting information from isotopomeric data using isotopic simulation coupled with optimization, we have included a simple example of the TCA cycle in *Saccharomyces uvarum*. This example introduces the basic process of extracting information from isotopomeric data and does not include many details in the modeling process such as sensitivity analysis and a through discussion of the flux parameters found. Judicious analysis of this system will require a separate publication.

The metabolic system is given in Figure 1 and was optimized using a non-linear constraint optimizer [72] using data collected by Paalme *et al.* [73]. We optimize a subset of their data where they performed a step change to fully labeled acetate while feeding yeast a mixture of glucose and acetate. Paalme *et al.* [73] measured ^13^C NMR absolute and conditional enrichments from the carbon skeleton of proteinogenic amino acids harvested and hydrolyzed at isotopic steady state. This excludes the optimization of pool sizes so they have all been set to be equal to simplify simulation, and all comparisons to measured data were made at the last time point simulated after all isotopic dynamics reached steady state.

We have included measurements of the rate of biomass production from all TCA metabolites in Figure 1 to constrain the net fluxes that exit the system. These net fluxes include all biomass production, including production of amino acids, nucleic acids, and lipids, however, only amino acids are included in the metabolic scheme since it was their isotopomers that were used to constrain the isotopic steady state. By not constraining the metabolic system explicitly using the biomass production rates the optimizer is given more flexibility to find better solutions by roaming around the full flux parameter space.

The optimization was carried out with the following reactions set to be bi-directional: malate dehydrogenase (EC 1.1.1.37), fumerase (EC 4.2.1.2), citrate synthase (EC 2.3.3.1), and the three transport reactions for oxaloacetate, pyruvate, and acetyl-coenzyme A. All reactions involving carbon dioxide, except for the bi-directional production of bicarbonate via carbonic anhydrase (EC 4.2.1.1), were set to be uni-directional.

**Figure 4.**
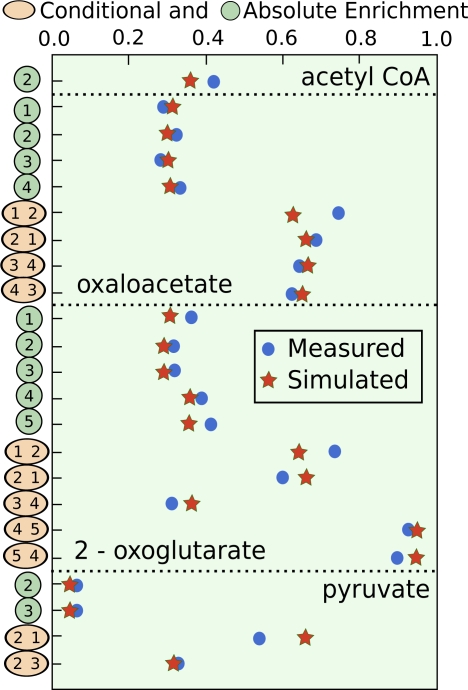
Optimization of example system with absolute and conditional ^13^C NMR data. Simulated points are marked with stars and measured data are marked with circles. Absolute enrichments are written with one carbon label, and conditional enrichments have a second carbon label. Conditional enrichment is the probability of ^13^C enrichment in the first carbon when the second carbon is a ^13^C.

By starting at a large number of plausible starting points selected at random over the range of the free flux parameters, the optimizer always settled on one single optimal solution and occasionally stopped at a few other local optima that did not reproduce the data very well. Changing the weighting of measured data points within the optimizer and excluding one or two at random did not significantly change the optimal solution found as this solution matched all available data quite well. The optimal fit to the isotopomeric data is given in Figure 4. It is immediately seen that the fit between the NMR data and the model predictions is very good. This means that this metabolic system can adequately account for the observed labeling pattern and no important elements of the metabolic system are missing. With regards to net fluxes, the optimal fit matches that found in [73].

With respect to bi-directional reactions, malate dehydrogenase was found to be very bi-directional with $\frac{v_{5f}}{v_{5r}}=1.3$, while the ratio for fumerase $\frac{v_{4f}}{v_{4r}}=575.0$. The transport of pyruvate was found to be quite reversible with $\frac{v_{200f}}{v_{200r}}=1.2$, while the transport of acetyl-coenzyme A was much less reversible with $\frac{v_{100f}}{v_{100r}}=12.4$, and the transport of oxaloacetate was found to be essentially uni-directional.

The pyruvate fit was the least perfect and the fit required the pyruvate transporter (R200) to be bidirectional. This may be telling us that the assumption that mitochondrial pyruvate is the sole precursor for Ala production is not entirely true, although at least some production of Ala from mitochondrial pyruvate is required to fit the data. Ala is produced from cytosolic pyruvate during fermentative growth so it is possible that both mitochondrial and cytosolic pyruvate act as precursors for Ala production, but this must be confirmed with additional data and future simulations possibly with the inclusion of an additional compartmentalized pool.

Pyruvate is a metabolite that participates in a large number of intersecting central metabolic pathways, typically has a low intra-cellular concentration, and has been observed to exhibit multiple cytoplasmic compartments along with mitochondrial compartmentalization [71, 74]. This hub metabolite may be compartmentalized in a more complicated way than has been supposed and should be studied with a larger data set containing dynamic isotopic transients.

**Figure 5.**
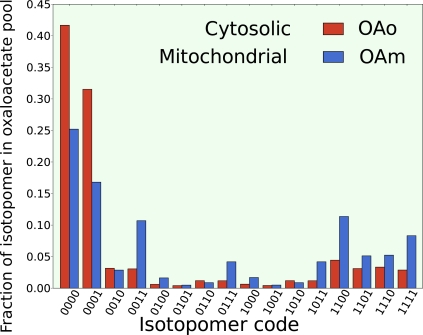
Simulated steady state isotopomer distribution of mitochondrial and cytosolic oxaloacetate. Since the isotopomers differ between compartments comparing the simulation with measured data can help determine the functional location of biosynthesis reactions.

The steady state isotopomer profiles of the cytosolic and mitochondrial pools of oxaloacetate are given in Figure 5. The labeling pattern in each compartment is quite different and has important implications for the origin of Asp biosynthesis as discussed by Paalme *et al.* [73]. These simulations support the previous findings that Asp synthesis originates from mitochondrial oxaloacetate since no adequate set of net flux and exchange flux parameters could be found that give a steady state isotopomer profile for cytosolic oxaloacetate that matched with the measured enrichments in the respective carbons in Thr and Ile [73].

To make the transient of this optimization quantitative we would have to include slow bi-directional exchange with storage compounds, since this has been found to dramatically influence the time scale of isotopic dynamics. The isotopic dynamics of TCA cycle metabolites such as 2-oxoglutarate, succinate, fumerate, glutamate, and aspartate, are all influenced by reversible aminotransferase reactions that transfer amino groups from α-amino acids to α-keto acids [12]. This makes the isotopic dynamics in the TCA cycle on the same temporal order of magnitude as reaching steady-state isotopomer labeling in the biomass. Accurate simulation of short time TCA dynamics requires a long term dynamic component that can only be quantified with labeling data from a long labeling experiment. Without accurate steady state labeling data, the interpretation of short term labeling experiments is difficult [75].

## 6. Conclusions

We have shown that dynamic isotopic transients reveal important insights into the operation of metabolic networks, including the bi-directionality of enzyme and transport reactions, and the compartmentalization of metabolites, including localized compartmentalization not separated by a membrane barrier and that caused by diffusional restrictions. Our optimization of the TCA cycle illustrates that using dynamic isotopic models does not complicate the analysis of steady state isotopomeric data if the transient part of the simulation is excluded, and the possibility for additional insight with the inclusion of only a small amount of transient data should not be overlooked. Models that make use of isotopic transient data are expected to become increasingly important as steady state isotopomeric models currently struggle with the realities of compartmentalization.

The predicted rise in the use of dynamic models is supported by the rapid development of analytical techniques to measure both isotopomeric transients and the kinetics of individual reactions *in vivo*. Numerical tools are also developing rapidly, however the current state of dynamic modeling continues to grapple with the difficulties of compartmentalization. Teasing out the details of compartmentalization using dynamic models involves the addition of more parameters. When introducing such parameters, the structural identifiability of the model must be preserved so that biological insight can be extracted from the measured data. This is a challenge for large metabolic systems and can only be accomplished by including as much information as possible to constrain the trajectories of the model solution. Examples include thermodynamic constraints, constraints on the pool sizes, integration of known kinetic information, and the fitting of isotopomeric data from as many experiments as possible.

Although a vast amount of kinetic detail is required to build predictive kinetic models, their use within isotopic transient models is expected to improve and expand phenomenological MFA. It is hoped that fundamental kinetic studies will once again become a funding priority and through their continuation support the use of kinetic schemes within realistically sized metabolic models, since the marriage of kinetics and MFA is predicted to become an ever increasingly important tool in systems biology.

## Figures and Tables

**Table 1.** Metabolite abbreviations within each compartment.

**Metabolite**	Abbreviation	
	Cytosolic	Mitochondrial
acetate	ACo	
acetyl-CoA	AcCoAo	AcCoAm
pyruvate	PYo	PYm
PY biomass precursor	PBm	
citrate/isocitrate		CIm
oxaloacetate	OAo	OAm
succinate		SUm
malate		MAm
2-oxoglutarate		OGm
